# Endoscopic hand-suturing training model

**DOI:** 10.1055/a-2282-9977

**Published:** 2024-04-03

**Authors:** Taku Morita, Ken Ohata, Toshihiro Araki, Makoto Koda, Kazuta Fukumori, Yohei Minato, Takumi Kawaguchi

**Affiliations:** 1 38636 Department of Gastroenterology, Omuta City General Hospital, Omuta, Japan; 2 Department of Gastroenterology, NTT Medical Center Tokyo, Tokyo, Japan; 3 200699 Department of Medichine, Division of Gastroenterology, Kurume University Hospital, Kurume, Japan

Endoscopic hand-suturing (EHS) is a novel suturing method that allows optimal and secure intraluminal suturing 1 2 . On the other hand, the procedure is reported to be complicated, difficult, and time-consuming 3 4 . At present, there is no appropriate training model for it and no established method for learning it.

We have developed a training sheet we called SuTURE (Suture Trainer Using Re-usable Elastomer; Kotobuki Medical Inc., Saitama, Japan) for EHS by applying the G-Master, which was initially developed as a training model for gastric endoscopic submucosal dissection (ESD) ( Fig. 1 ). SuTURE has a two-layer structure of elastomer, a polymer material that exhibits rubber-like elasticity, and has a virtual mucosal defect with a major diameter of 40 mm on the upper sheet and a gusset at the edge to reproduce the sensation of applying a needle similar to that of a real case ( Fig. 2 ).

**Fig. 1:**
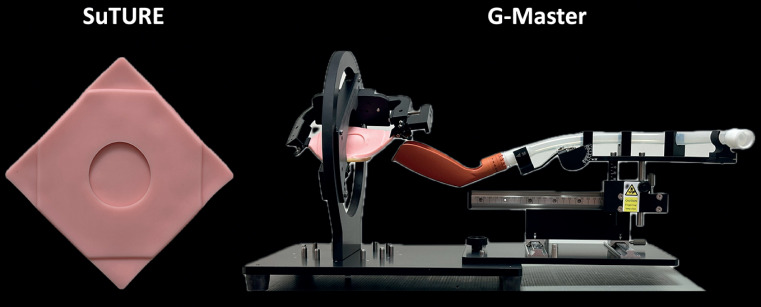
Suture Trainer Using Re-usable Elastomer (SuTURE) & G-Master (training model for endoscopic submucosal dissection (ESD))

**Fig. 2:**
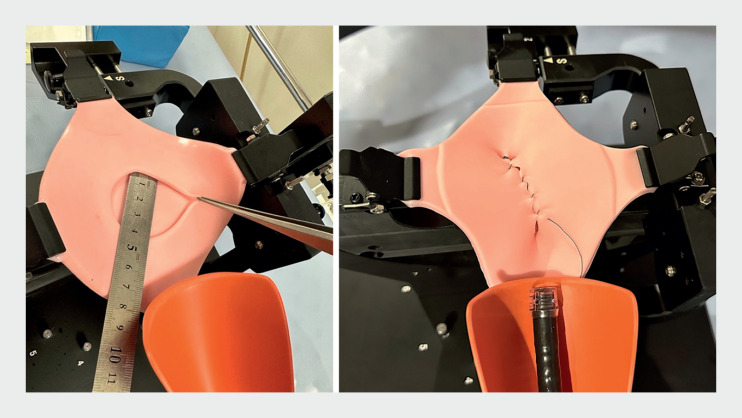
Two-layer sheet with gussets for 40-mm virtual post-ESD ulcer.

It is also strong enough to practice suture closure without tearing when pulled with a thread. The difficulty of the EHS operation depends on the angle at which the lesion is confronted. The G-Master is an ESD training model characterized by the ability to freely set the angle at which the scope faces the lesion. EHS training was performed by fixing the scope and SuTURE in various positions, such as perpendicular, anterior wall side, and superior wall side ( Fig. 3 , Video 1 ).

**Fig. 3:**
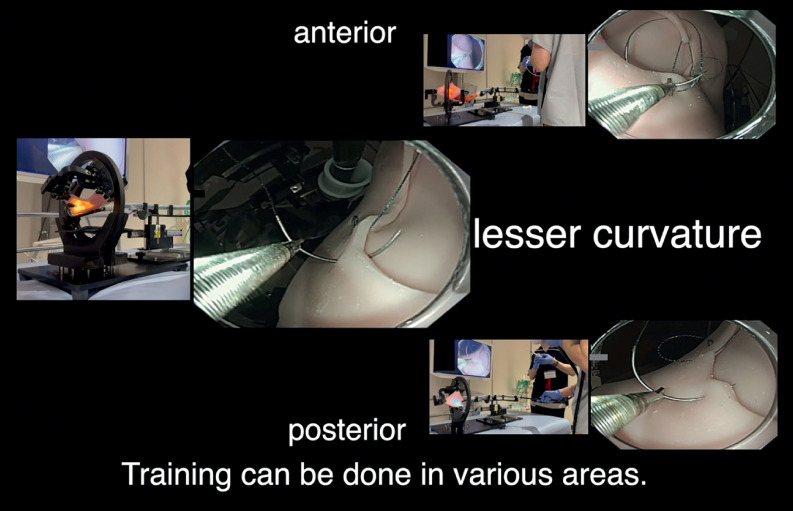
You can train with various body parts that can be set with G-Master.

Features of Suture Trainer Using Re-usable Elastomer (SuTURE) and actual training.Video 1

This virtual lesion does not use biological material and does not require a dedicated room or endoscope. It is easy to prepare and can be disposed of as regular trash after training.

At present, there is no EHS specialized training model, but EHS requires technical mastery. This training model could contribute to EHS proficiency.

Endoscopy_UCTN_Code_TTT_1AU_2AB

## Notes

Endoscopy E-Videos https://eref.thieme.de/e-videos
*E-Videos* is an open access online section of the journal *Endoscopy* , reporting on interesting cases and new techniques in gastroenterological endoscopy. All papers include a high-quality video and are published with a Creative Commons CC-BY license. Endoscopy E-Videos qualify for HINARI discounts and waivers and eligibility is automatically checked during the submission process. We grant 100% waivers to articles whose corresponding authors are based in Group A countries and 50% waivers to those who are based in Group B countries as classified by Research4Life (see: https://www.research4life.org/access/eligibility/ ). This section has its own submission website at https://mc.manuscriptcentral.com/e-videos .
